# The features of muscle activity during chair standing and sitting motion in submerged condition

**DOI:** 10.1371/journal.pone.0220602

**Published:** 2019-08-08

**Authors:** Koichi Kaneda

**Affiliations:** Faculty of Advanced Engineering, Chiba Institute of Technology, Narashino, Chiba, Japan; Northwestern University Feinberg School of Medicine, UNITED STATES

## Abstract

This study aimed to measure muscle activity and motion kinematics during chair-based exercises under submerged and non-submerged conditions. Twelve healthy men performed chair-based standing and sitting movements. Surface electrodes were attached at the tibialis anterior, gastrocnemius, rectus femoris, biceps femoris, rectus abdominis, and erector spinae. The ankle, knee, and hip joint angles and forward inclination angle of the trunk segment in the sagittal plane were calculated. The mean muscle activities during both movements in the submerged condition for the entire motion were lower than those in the non-submerged condition except in the tibialis anterior and biceps femoris during the sitting movement (in the standing exercise, rectus femoris: 14.1% and 5.2%; and erector spinae: 18.3% and 13.6% in non-submerged and submerged conditions, respectively; and in the sitting exercise, rectus femoris: 12.1% and 4.5% and erector spinae: 12.9% and 9.9% in the non-submerged and submerged conditions, respectively). However, the integrated muscle activity in submerged conditions was similar or higher to that in non-submerged conditions during both movements, except for the rectus femoris. This was mainly due to the increased duration of motion (44.3% and 39.9% longer for standing and sitting exercises in submerged conditions, respectively, compared with non-submerged conditions). The hip joint flexion at the beginning and end of movement and forward inclination angles of the trunk segment at the beginning of the movement in the submerged condition were larger than those in the non-submerged condition during both movements (hip: 126.1° and 111.5° at the beginning, 182.3° and 178.4° at the end and trunk: 2.7° and 17.4° at the beginning in non-submerged and submerged conditions for the standing exercise, respectively; hip: 182.4° and 178.0° at the beginning, 125.9° and 111.1° at the end and trunk: 2.2° and 16.9° at the end in non-submerged and submerged conditions for the sitting exercise, respectively). Reduced or similar muscle activity but similar or higher muscular effort was observed in the submerged condition for all the muscles except the rectus femoris, with the upper body inclined forward. These findings could have beneficial implications for the prescription of exercise and rehabilitation regimens.

## Introduction

Chair-based standing and sitting movements are among the most fundamental activities of daily living (ADLs) besides walking [1, 2]. Deterioration in the ability to perform these actions is associated with muscular weakness and loss of balance control [3, 4]. To date, many biomechanical studies have been conducted to assess chair-based standing and sitting movements using force plates [1, 5], motion analysis [2, 5, 6], inertial sensors [7], and electromyography (EMG) [1, 2, 5, 8]. These studies aimed to clarify the kinematic and kinetic mechanisms of these movements and most were performed in rehabilitation and functional exercise settings.

Submerged exercise has become popular for rehabilitation and functional training. In the submerged state, buoyancy reduces stress associated with gravity during exercise, while water resistance increases the driving force required to move the limbs by human [9]. Recently, many studies have reported the characteristics of submerged locomotion, which were mostly conducted for walking, using different biomechanical measurement methods such as force plates [10–12], motion analysis [13, 14], inertial sensors [15], and EMG [13, 14, 16]. These studies reported significant differences in the characteristics of movements performed when submerged and not submerged.

While many biomechanical studies on walking in the submerged state have been conducted, little has been done to investigate movements involved in standing and sitting from a chair despite these movements being used in aquatic rehabilitation and functional exercise programs [17–19]. As long as the knowledge of the author, only Cuesta-Vargas et al. [17] reported the differences in muscle activity during movements involved in standing from a chair between the submerged and non-submerged states. They detected higher muscle activity in the trunk muscles in the submerged state. However, the study involved only muscle activity measurement, without motion analysis. When assessing the kinesiology of submerged exercise, motion analysis is considered essential. In addition, movements involved in sitting on a chair from a standing position were not assessed in their study [17]. It is important to clarify the characteristics of not only standing but also sitting movements because both movements are commonly conducted in daily life. Research into this area will be useful for determining appropriate rehabilitation and functional training prescriptions.

With the above in mind, the purpose of this study was to measure muscle activity and movement kinematics during chair-based standing and sitting movements with the participant either submerged or not submerged. The hypothesis was that the muscle activity of the lower limb would be reduced in submerged conditions compared with non-submerged conditions. With regards to motion kinematics, we expected to find a similar pattern but a somewhat more flexed posture of the lower-limb joints and trunk segment in submerged conditions compared with non-submerged conditions during both chair standing and sitting movements, as is seen during walking [10, 13, 14, 20].

## Methods

### Participants

Twelve healthy men participated in the present study. Their means ± standard deviations (SDs) of age, height, weight, and body mass index were 29.8 ± 8.8 years, 174.3 ± 5.0 cm, 67.8 ± 5.5 kg, and 22.3 ± 1.9 kg∙m-2, respectively. They had no orthopedic diseases that could affect chair-based standing/sitting. We obtained written informed consent from all participants before conducting the study. The Human Ethics Committee of Chiba Institute of Technology approved the protocol.

### Design and procedures

The experiment was conducted using a made-to-order water tank (Japan Aqua Tec Co., LTD, Japan) with a glass window on one side for exercises in the submerged condition and a concrete floor adjacent to the water tank for exercises in the non-submerged condition. Participants sat on a steel box without a back rest and performed five consecutive sit-to-stand and stand-to-sit movements with three sets. The height of the steel box was 40 cm, as described in a previous study [4]. The exercise intensity was set at a self-selected comfortable pace [13, 14, 21]. In the submerged condition, the depth of the swimming pool was set at 1 m, where the depth from the water's surface to the chair's sitting surface was 60 cm, equivalent to the clavicle level of each participant. The same box was used for the non-submerged exercises. The men sat in a comfortable position with their arms folded in front of their bodies. This arm position was maintained during the movements. The foot and seat positions were determined by each participant according to comfort. The order in which the submerged and non-submerged exercises were performed was randomized in each participant. The water and air temperatures were 31.2 ± 0.9°C and 22.5 ± 2.5°C, respectively.

### Measures

The muscle activity of the right side of the tibialis anterior (TA), medial head of the gastrocnemius (GAS), rectus femoris (RF), long head of the biceps femoris (BF), rectus abdominis (RA), and erector spinae (ES) was measured during the movements. A wireless EMG sensor (LP-WS1221, Logical Product Corp, Japan) was placed on the muscle belly and the data were collected at a 1-kHz sampling rate. The attachment locations were determined based on the Anatomical Guide for EMG [22]. The skin cuticle was removed using skin pure (Skin Pure; Nihon Kohden Corp., Japan) and the skin was cleaned with alcohol wipes. The distance between the two metal electrodes of the wireless EMG sensor was 2 cm, with the reference electrode placed between the two. The wireless EMG sensors were water-proofed by attaching a transparent film (Million Aid Dressing Tape; KYOWA Limited, Japan) and foam pads (Foam Pad; Nihon Kohden Corp., Japan).

Participants performed an isometric maximal voluntary contraction (MVC) two times for each muscle while not submerged, before the chair-based exercise. The MVC trials in each muscle were based on the Muscle Testing of the Anatomical Guide for EMG [22]. To evaluate MVC of the TA, participants sat on the chair with the knee joint flexed at about 90° with the sole of the foot on the ground. They were then asked to produce a force to create dorsi-flexion of the ankle joint. The foot was supported manually by the experimenter to obtain isometric contraction. For assessment of the GAS, participants stood upright and produced a force to move into a tiptoe position. The shoulder was supported manually by the experimenter. For the RF and BF, participants sat on the chair as for the MVC of TA measurement, and were asked to produce a force to create knee extension for the RF, and knee flexion for the BF. Manual support was provided by the experimenter to the ankle for RF and the heel for BF. With regards to the RA, participants lay in the supine position with the knee flexed at about 90°, with the sole of the foot on the ground and hands crossed at the back of the head. They were then asked to produce a force to create trunk flexion. The experimenter sat on the participant’s knees and supported the elbows. The participants lay in a prone position with hands crossed at the back of the head for assessment of ES, then were asked to produce a force to create trunk extension. The scapula was supported by the experimenter. Before each MVC measurement, the participants were familiarized with the MVC measurement procedure. The duration of the MVC tests was 5 s for each muscle. A sufficient period of rest was allowed between the two MVC measurements.

The EMG data of the MVC tests and chair-based exercises were filtered using fourth-order high and low-pass filters with 10-Hz and 500-Hz cut-off frequencies, respectively. The root mean square (RMS) was then calculated from the entire recording in 100-ms windows. The peak 1-s RMS value of MVC in each muscle was treated as the 100% value. The higher value from two MVC trials was adopted as the 100% value. Then, the percentages of MVC (%MVC) during chair-based exercises were computed in each muscle.

A motion capture system (VENUS 3D; Nobby Tech. Ltd, Japan) synchronized with the EMG sensor unit was placed on the right side of the participant to capture the chair and participant during standing/sitting in both conditions. The sampling rate of the motion capture system was 100 Hz. A wireless active marker (Kirameki; Nobby Tech. Ltd, Japan) was attached at the fifth metatarsal head, lateral malleolus, lateral epicondyle of the femur, greater trochanter, midpoint on the iliac spine, and acromion. All markers were placed on the right side of the participant.

### Statistical analysis

The marker position was filtered at 3 Hz [5, 23] using a fourth order low-pass filter. The foot (the fifth metatarsal head to lateral malleolus), lower leg (the lateral malleolus to the lateral epicondyle of the femur), upper leg (the lateral epicondyle of the femur to the greater trochanter), pelvis (the greater trochanter to the midpoint on the iliac spine), and trunk (the midpoint on the iliac spine to the acromion) segments were then defined. From these defined segments, the ankle, knee, and hip joint angular displacement values in the sagittal plane and the trunk inclination angle with respect to the vertical axis in the sagittal plane were calculated. In the present study, positive values indicated ankle plantarflexion, knee extension, hip extension, and forward inclination of the trunk segment.

The EMG and motion capture data were collected from the beginning to the end of each sit-to-stand and stand-to-sit movement based on the angular velocity of the trunk segment. The threshold value was set at < or > 0.087 rad∙s-1 (5° per second) according to a previous study [7, 21]. The collected data were divided into three phases: phase 1 (P1) comprised the beginning of the sit-to-stand movement to the beginning of knee joint extension (the threshold of the knee joint angular velocity was set at 0.087 rad∙s^-1^ [5° per second]), phase 2 (P2) comprised the beginning of knee joint extension to the reversal of trunk segment flexion–extension, and phase 3 (P3) comprised the reversal of trunk segment flexion–extension to the end of sit-to-stand in the sit-to-stand movement. In the stand-to-sit movement, P1 comprised the beginning of stand-to-sit to the reversal of trunk segment flexion–extension, P2 comprised the reversal of trunk segment flexion–extension to the end of knee joint flexion (threshold of knee joint angular velocity was the same), P3 comprised the end of knee joint flexion to the end of stand-to-sit. These phase distinction methods were referred from a previous study [24]. After data collection, the mean values of EMG (mEMG) for the entire motion and each phase and the integrated value of EMG (iEMG) for the entire motion were calculated. The iEMG value was calculated after the %MVC wave was computed. The iEMG values are therefore expressed in % MVIC∙s. For the motion data, the angle of each joint and trunk inclination at the beginning and ending of each moment and range of motion (ROM) during each movement for the entire motion and each phase were computed. The ROM was calculated as the difference between the maximum and minimum values. The data of the third time within consecutive standing/sitting motion in the second and third sets were averaged. The duration of P2 in the second trial of the sit-to-stand movement was computed to be negative for one participant. For this participant, only the data of each phase of the third trial was utilized. The data were expressed as mean ± standard deviations (SDs). The Kolmogorov-Smirnov test was applied to evaluate the normality of the data and equality was examined using Bartlett's test. When the data were treated as non-parametric by these tests, the Wilcoxon signed-rank test was used to detect any differences between the two conditions. A paired t-test was applied when the data were parametric. The significance level was set at p < 0.05.

## Results

The durations for the sit-to-stand movement were 1.85 ± 0.31 s in the non-submerged condition and 2.67 ± 0.43 s in the submerged condition for the entire motion. The durations of each phase in non-submerged and submerged conditions were P1: 0.53 ± 0.16 s and 0.87 ± 0.37 s, P2: 0.34 ± 0.07 s and 0.38 ± 0.17 s, and P3: 0.98 ± 0.16 s and 1.41 ± 0.20 s, respectively. In the stand-to-sit movement, durations in non-submerged and submerged conditions were 2.01 ± 0.25 s and 2.82 ± 0.30 s for the entire motion, P1: 0.94 ± 0.15 s and 1.40 ± 0.25 s, P2: 0.50 ± 0.15 s and 0.75 ± 0.30 s, and P3: 0.57 ± 0.15 s and 0.67 ± 0.31 s, respectively. Significantly longer durations (p < 0.05) were detected in the submerged condition than in the non-submerged condition during the sit-to-stand movement for the entire motion (44.3%), P1 (62.3%), and P3 (44.1%). In the stand-to-sit movement, significantly longer durations (p < 0.05) were recorded in the submerged than non-submerged condition for the entire motion (39.9%), P1 (48.3%), and P2 (49.1%).

**Fig 1:**
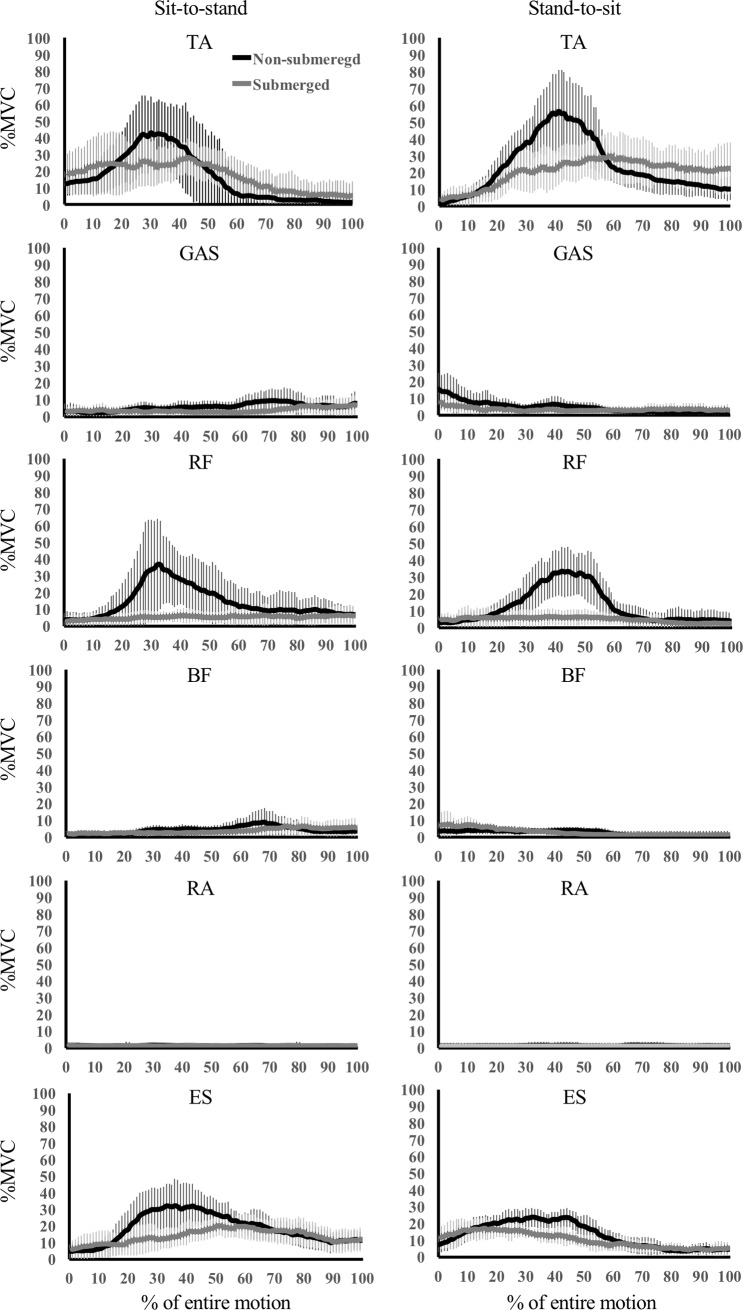
The time course pattern of each muscle during sit-to-stand and stand-to-sit movements in the submerged and non-submerged conditions. TA: tibialis anterior, GAS: medial head of the gastrocnemius, RF: rectus femoris, BF: long head of the biceps femoris, RA: rectus abdominis, ES: erector spinae MVC: maximum voluntary contraction.

The mEMG values of each muscle during each movement are shown in Table 1, and the ensemble curves of EMG are depicted in Fig 1. The mEMG values of all muscles were significantly lower (p < 0.05) in submerged conditions compared with non-submerged conditions for the entire motion of the sit-to-stand movement, except for the TA muscle. Significantly lower mEMG values were observed in submerged conditions compared with non-submerged conditions during the sit-to-stand movement in the RF, RA, and ES muscles for P1; in the RF, BF, RA, and ES muscles for P2; and the GAS, RF, and RA muscles for P3. The mEMG value of the TA muscle during the sit-to-stand movement was found to be higher in submerged than in non-submerged conditions for P3. Significantly lower mEMG values (p < 0.05) were seen in the submerged condition than in the non-submerged condition for all muscles for the entire motion in the stand-to-sit movement, except for the TA and BF muscles. For each phase, significantly lower mEMG values were calculated for submerged compared with non-submerged conditions during the stand-to-sit movement in the TA, GAS, RF, RA, and ES muscles for P1; in the RF, BF, RA, and ES muscles for P1; and RF and RA muscles for P3. The mEMG values were significantly higher in submerged conditions than in non-submerged conditions during the stand-to-sit movement in the BF muscle for P1, and the TA and GAS for P3.

**Table 1:** The mEMG values during sit to stand and stand to sit motion in both conditions for the entire motion and each phase.

Through the movement	Sit-to-stand			Stand-to-sit		
	Non-submerged	Submerged	%change from non-submerged	Non-submerged	Submerged	%change from non-submerged
TA (%)	16.5 ± 8.3	17.2 ± 7.2	4.6	23.9 ± 7.7	20.4 ± 9.1	-14.5
GAS (%)	5.7 ± 1.9	3.7 ± 1.7 *	-35.1	4.8 ± 2.1	3.3 ± 1.7 *	-30.8
RF (%)	14.1 ± 9.6	5.2 ± 3.2 *	-63.1	12.1 ± 4.5	4.5 ± 3.3 *	-63.1
BF (%)	4.1 ± 1.9	3.4 ± 1.6 *	-17.9	2.6 ± 0.7	2.9 ± 1.0	14.6
RA (%)	1.3 ± 0.5	1.1 ± 0.4 *	-14.8	1.2 ± 0.5	1.0 ± 0.5 *	-11.5
ES (%)	18.3 ± 4.6	13.6 ± 3.9 *	-25.9	12.9 ± 3.1	9.9 ± 3.4 *	-23.6
P1	Sit-to-stand			Stand-to-sit		
	Non-submerged	Submerged	%change from non-submerged	Non-submerged	Submerged	%change from non-submerged
TA (%)	21.9 ± 11.0	23.7 ± 14.3	8.3	26.9 ± 9.1	15.5 ± 6.4*	-42.6
GAS (%)	3.1 ± 2.0	3.3 ± 2.6	8.0	7.5 ± 3.7	3.5 ± 1.2 *	-53.6
RF (%)	9.0 ± 8.2	3.5 ± 2.6 *	-61.3	15.1 ± 6.8	5.1 ± 4.5 *	-66.3
BF (%)	1.7 ± 0.8	2.0 ± 1.8	14.5	3.6 ± 1.0	4.5 ± 2.0 *	26.7
RA (%)	1.3 ± 0.5	1.1 ± 0.7 *	-12.2	1.2 ± 0.5	1.0 ± 0.4 *	-12.2
ES (%)	12.1 ± 5.6	9.3 ± 6.1 *	-23.5	18.5 ± 4.0	13.9 ± 4.0 *	-24.9
P2	Sit-to-stand			Stand-to-sit		
	Non-submerged	Submerged	%change from non-submerged	Non-submerged	Submerged	%change from non-submerged
TA (%)	39.0 ± 20.1	28.2 ± 15.1	-27.6	29.0 ± 10.8	26.8 ± 12.0	-7.5
GAS (%)	5.5 ± 2.8	4.0 ± 3.1	-26.3	3.1 ± 1.7	3.1 ± 2.3	-1.3
RF (%)	30.2 ± 19.9	5.6 ± 3.2 *	-81.5	15.2 ± 6.2	4.4 ± 2.9 *	-71.1
BF (%)	4.8 ± 1.7	2.4 ± 1.0 *	-50.5	2.3 ± 1.2	1.4 ± 0.5 *	-37.5
RA (%)	1.4 ± 0.6	1.0 ± 0.5 *	-25.5	1.2 ± 0.5	1.0 ± 0.5 *	-13.9
ES (%)	32.2 ± 11.6	17.7 ± 5.4 *	-45.1	11.2 ± 3.7	6.6 ± 2.6 *	41.3
P3	Sit-to-stand			Stand-to-sit		
	Non-submerged	Submerged	%change from non-submerged	Non-submerged	Submerged	%change from non-submerged
TA (%)	6.4 ± 9.0	11.3 ± 9.2*	77.2	13.3 ± 6.5	21.8 ± 12.1 *	64.4
GAS (%)	7.4 ± 4.0	4.5 ± 2.6 *	-39.3	1.8 ± 0.9	3.1 ± 2.8 *	66.5
RF (%)	11.0 ± 8.7	5.6 ± 4.4 *	-48.8	4.4 ± 5.7	2.0 ± 1.7 *	-55.8
BF (%)	5.3 ± 3.1	4.8 ± 3.3	-10.0	1.1 ± 0.4	1.4 ± 0.7	30.3
RA (%)	1.2 ± 0.5	1.1 ± 0.4 *	-8.6	1.2 ± 0.5	1.1 ± 0.5 *	-10.6
ES (%)	16.8 ± 5.5	16.1 ± 5.8	-3.8	4.7 ± 2.6	4.4 ± 2.5	-5.4

*: Significant difference between two conditions (p < 0.05).

P1: phase 1, P2: phase 2, P3: phase 3

TA: tibialis anterior, GAS: medial head of the gastrocnemius, RF: rectus femoris, BF: long head of the biceps femoris, RA: rectus abdominis, ES: erector spinae

The iEMG values are expressed in Table 2. Significantly higher iEMG values (p < 0.05) in the submerged condition than those in the non-submerged condition were detected in the TA and RA muscles during the sit-to-stand movement, whereas significantly lower iEMG values (p < 0.05) were seen in the RF (44.4% lower) muscle in the submerged condition than in the non-submerged condition. During the stand-to-sit movement, significantly lower iEMG values in the RF muscle and higher iEMG values in the BF and RA muscle (p < 0.05) were detected in the submerged condition than in the non-submerged condition.

**Table 2:** iEMG values during sit to stand and stand to sit motion in both conditions.

	Sit-to-stand			Stand-to-sit		
	Non-submerged	Submerged	%change from non-submerged	Non-submerged	Submerged	%change from non-submerged
TA (%)	30429.6 ± 17826.5	45827.5 ± 17540.1 *	50.6	47349.9 ± 15620.4	56645.2 ± 27569.9	19.6
GAS (%)	10591.7 ± 4246.6	10068.8 ± 4750.1	-4.9	9777.8 ± 4887.1	9530.1 ± 5557.2	-2.5
RF (%)	25209.7 ± 13716.1	14009.4 ± 9017.8 *	-44.4	24069.6 ± 8619.0	12280.0 ± 8391.6 *	-49.0
BF (%)	7557.2 ± 3745.4	9516.3 ± 5399.7	25.9	5096.8 ± 1451.9	8267.3 ± 3019.2 *	62.2
RA (%)	2345.8 ± 1030.4	2864.5 ± 1148.1 *	22.1	2370.8 ± 1079.3	2947.6 ± 1410.3 *	24.3
ES (%)	33084.0 ± 6416.9	36222.2 ± 9820.2	9.5	25757.7 ± 6722.8	26973.7 ± 7212.6	4.7

*: Significant difference between two conditions (p < 0.05).

TA: tibialis anterior, GAS: medial head of the gastrocnemius, RF: rectus femoris, BF: long head of the biceps femoris, RA: rectus abdominis, ES: erector spinae

The unit of iEMG is %MVIC ∙ s by right because the iEMG was calculated after %MVC values were computed.

**Fig 2:**
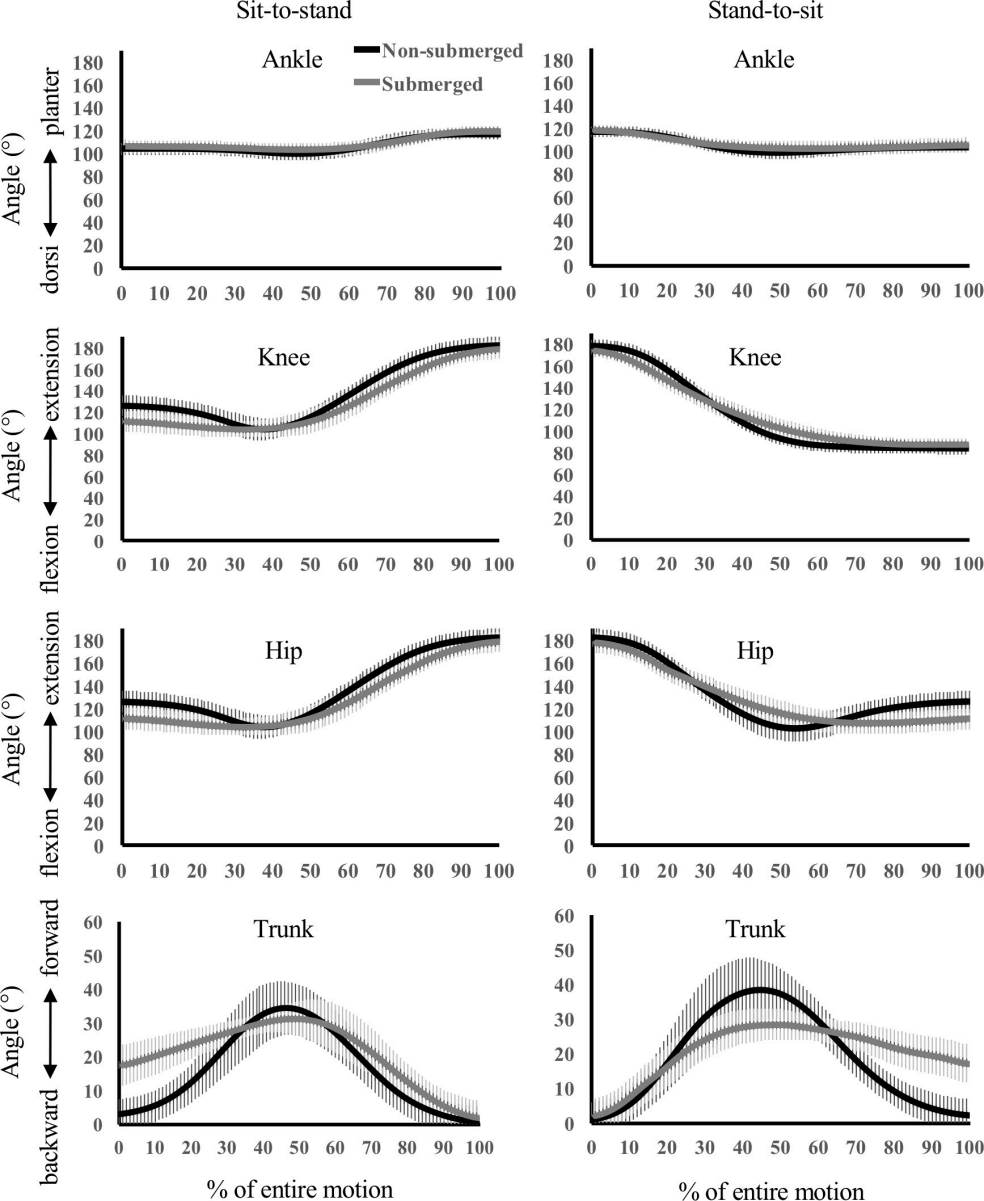
The time course pattern of each joint and trunk inclination angle during sit-to-stand and stand-to-sit movements in the submerged and non-submerged conditions.

The mean angles at the moment of beginning and ending each movement in both conditions shown in Table 3. The ensemble curves for each kinematic parameter are depicted in Fig 2. Significantly lower values (p < 0.05) in the submerged condition than in the non-submerged condition were seen in the ankle joint for ROM in the stand-to-sit movement. In the knee joint, significantly higher values (p < 0.05) in the submerged condition than in the non-submerged condition were detected at the beginning of the sit-to-stand movement and at the end of the stand-to-sit movement. In addition, significantly lower values (p < 0.05) during the submerged condition than during the non-submerged condition were seen at the end of the sit-to-stand movement and the beginning of the stand to sit movement, and for ROM in both movements. The hip joint showed significantly lower values (p < 0.05) in the submerged condition than in the non-submerged for the beginning, end, and ROM of both movements. With respect to trunk inclination angle, significantly higher values (p < 0.05) in the submerged condition than in the non-submerged condition were seen at the beginning of the sit-to-stand movement and at the end of the stand-to-sit movement. The ROM of the trunk inclination angle was significantly lower (p < 0.05) in the submerged condition than in the non-submerged condition.

**Table 3:** Each joint and trunk inclination angle at beginning and ending moment and ROM during sit to stand and stand to sit motions in both conditions.

		Sit-to-stand		Stand-to-sit	
		Non-submerged	Submerged	Non-submerged	Submerged
Ankle (°)	Beginning	103.8 ± 4.9	105.8 ± 5.9	117.3 ± 4.5	118.3 ± 4.5
	Ending	117.2 ± 4.6	118.7 ± 4.7	103.8 ± 5.6	105.4 ± 6.6
	ROM	19.7 ± 4.3	18.4 ± 4.1	18.8 ± 5.3	16.9 ± 4.3 *
Knee (°)	Beginning	84.2 ± 5.0	87.4 ± 4.8*	178.4 ± 5.9	174.3 ± 6.1 *
	Ending	178.5 ± 5.9	175.4 ± 6.4 *	84.0 ± 5.4	87.6 ± 5.3 *
	ROM	94.8 ± 5.1	88.5 ± 5.0 *	94.5 ± 5.2	87.5 ± 4.8 *
Hip (°)	Beginning	126.1 ± 9.6	111.5 ± 9.9 *	182.4 ± 7.8	178.0 ± 8.9 *
	Ending	182.3 ± 7.8	178.4 ± 8.9 *	125.9 ± 9.5	111.1 ± 8.9 *
	ROM	80.4 ± 9.7	78.2 ± 10.5	81.4 ± 12.5	73.1 ± 12.1 *
Trunk (°)	Beginning	2.7 ± 4.5	17.4 ± 6.0 *	0.9 ± 4.9	1.9 ± 5.0
	Ending	0.4 ± 4.8	1.7 ± 5.0	2.2 ± 4.6	16.9 ± 5.6 *
	ROM	35.8 ± 7.8	31.5 ± 6.4	39.9 ± 9.9	27.2 ± 6.7 *

*: Significant difference between two conditions (p < 0.05).

The positive value indicates ankle planter flexion, knee extension, hip extension and forward trunk inclination.

ROM: range of motion

## Discussion

This is the first study to investigate muscle activity during both sit-to-stand and stand-to-sit movements with the participants submerged in water. To our knowledge, only one previous study [17] has investigated sit-to-stand movements in the submerged condition. However, this previous study reported only muscle activity, whereas we used motion analysis in addition to muscle activity measurement. In addition, no previous studies have investigated muscle activity during the stand-to-sit movement using motion analysis in submerged conditions. Exercising while sitting on a chair in submerged conditions is intended to simulate real world (non-submerged) conditions, and has been used as part of health maintenance and rehabilitation training [18, 19]. This study presents novel information on the muscle activity and kinematics of chair-based exercises in submerged conditions, which may be beneficial during the design and prescription of health and rehabilitation programmes.

The present study divided exercises into sit-to-stand and stand-to-sit movements. The duration for both movements in the non-submerged condition seemed reasonable when compared with the results of the study by Walaszek et al. [6] and some other previous studies [5, 7, 23]. In addition, the present study further separated each movement into three phases as has been suggested in a previous study [24]. When compared with data of the previous study [24], the proportions of the duration of the entire motion that were represented by each phase in the sit-to-stand movement were similar for P1 (27% in the previous study and 28.5% in the present study), although slightly longer in the present study (18.3%) than in the previous study (9%) for the duration of the P2. This difference may be attributable to the fact that participants of the previous study [24] were aged 65–76 compared with the 28.9 year mean age of participants of the present study, as the strategy of the standing motion would be different between ages due to the fitness level difference. In addition, the mEMG values of our study for the sit-to-stand movement were also similar to those of a previous study by Cuesta-Vargas et al. [17] except for the TA, RA, and ES in the submerged condition. Regarding the stand-to-sit movement, a few studies have been conducted in the non-submerged condition and mEMG values of the GAS and semitendinosus (ST) muscles reported by Walaszek et al. [6] were similar to those detected in the present study in the GAS and BF, where the role of the ST and BF was to flex the knee joint and extend the hip joint. Therefore, the data obtained in the present study appear to align with the results of previous studies on standing/sitting movements in the non-submerged condition.

Cuesta-Vargas et al. [17] compared trunk and lower extremity mEMG during sit-to-stand movements in the submerged condition and non-submerged condition. They reported significantly lower mEMG values in the TA muscle in the submerged condition than in the non-submerged, while the present study showed a non-significant difference for the entire motion. A significantly lower mEMG value was calculated for P1 in the stand-to-sit movement, and significantly higher mEMG values were calculated for P3 in both movements. The TA muscle activates not only ankle joint dorsiflexion but also tap and/or wiggle movements of the toe, and such motions cannot be detected in the ankle motion analysis used in the present study. The ensemble curve of the ankle joint appears very similar in both conditions, and almost no difference was observed in the kinematic values except for ROM in the stand-to-sit movement which was slightly lower in submerged than non-submerged conditions. Multiple factors would affect mEMG values particularly in the submerged condition, in which balance is continuously changing with movement due to buoyancy and water resistance. A previous study reported high intra- and inter-participant variability at the TA muscle during walking in the submerged condition [16]. Although the reason for the higher iEMG values in the submerged condition during sit-to-stand movements in the TA muscle could be the longer duration in the submerged condition than in the non-submerged condition, further detailed analysis is necessary to determine the behavior of the TA.

In the present study, mEMG values of the GAS muscle were significantly lower in submerged than non-submerged conditions for the entire motion of both movements, in the stand-to-sit movement for P1, and in the sit-to-stand movement for P3. A previous study [17] supports our results of mEMG of the entire movement with respect to the sit-to-stand movement, and we can conclude that the mEMG values were affected by buoyancy. The weight load is offset according to the level of immersion [11], resulting in reduced muscle load. However, no differences in the iEMG values were found between the two conditions for both movements. This is likely due to the longer duration of movement in the submerged condition compared with the non-submerged condition, which strongly affected the iEMG value.

The mEMG values of the RF muscle were significantly lower in the submerged condition than the non-submerged condition for the entire motion in both movements, which is in agreement with data of a previous study relating to the GAS muscle [17]. One reason for this is the reduction of muscle load due to buoyancy. In addition, the ROM was smaller in submerged conditions for the knee joint in both movements and the hip joint in the stand-to-sit movement, with larger forward inclination of the trunk segment when in a sitting position. These kinematic changes would imply that increased trunk forward-inclination, due to compensation for the forward translation of the center of buoyancy [20, 25, 26], contributed to reduction of the muscle load of the RF. The iEMG values were also significantly lower in the submerged condition than in the non-submerged condition in both movements, regardless of the increased duration. This may support the suggestion that there were not only buoyancy effects, but also postural changes (especially in the trunk segment) that affected muscle load of the RF in submerged conditions.

With respect to the BF muscle, a significantly lower mEMG value in the submerged condition than in the non-submerged condition was observed in the sit-to-stand movement, as reported by a previous study [17]. This was probably caused by buoyancy supporting the extension of the hip joint to move the body upward. Although the mEMG value of the BF muscle was identical for the entire motion in the two conditions of the stand-to-sit movement, higher mEMG values were observed for P1 and lower mEMG values for P2. During the stand-to-sit movement, the BF primarily acts to create knee joint flexion to move the body downward. The joint angle and ROM of the knee were more flexed and smaller, respectively, in the submerged condition compared with the non-submerged condition, which could explain the low mEMG value for the BF muscle. However, when the ensemble curve of the knee joint angle is examined, a slightly increased flexion can be seen in the submerged condition compared with the non-submerged condition during 0 to 30% of the entire motion. Furthermore, there was a slight increase in extension in the submerged condition compared with the non-submerged condition during 40 to 70% of the entire motion. These slight differences in knee joint angle curve may affect the mEMG values. The non-significant difference between iEMG values of both conditions in the sit-to-stand movement, and the higher values in the submerged compared with the non-submerged condition in the stand-to-sit movement, could simply be due to the increased duration in the submerged condition.

The mEMG value of the RA muscle in the present study showed fairly low activity in both movements and conditions, although significantly lower mEMG values were seen in the submerged condition than in the non-submerged condition during both movements. A previous study reported higher mEMG values in the submerged condition than in the non-submerged condition during the sit-to-stand movement [17]. In addition, the mEMG values of the ES muscle were lower in the submerged condition than in the non-submerged condition during both movements except for P3 in the present study, whereas the previous study [17] indicated higher values during the sit-to-stand movement in the submerged condition than in the non-submerged condition. One of the reasons for these discrepancies could be pace setting. In the previous study, the pace was set at 20 beats per min, whereas in the present study it was set according to the participant's level of comfort. Pace restriction may produce extra muscle activity for movement control particularly in the submerged condition, in which the buoyancy changes during movements, affecting posture control [17]. The large SDs in %MVC for the RA and ES muscles in the submerged condition in the previous study could indicate the effect of pace restriction. Another reason for the discrepancies could be that almost no trunk joint flexion motion was generated during the exercises of the present study. In the previous study [17], two significant EMG peaks were observed: one for trunk flexion and one moving the body upward. However; in the present study, there was no significant EMG peak for the RA muscle and only one gentle peak for the ES muscle. We detected greater flexion at the hip joint and a more inclined posture of the trunk segment at the beginning of the sit-to-stand movement in submerged compared with non-submerged conditions. This indicates that there was almost no trunk joint flexion motion around the beginning of the sit-to-stand movement in the submerged condition. A greater forward inclination of the trunk segment has also been observed in weightlessness conditions [25], and in submerged walking and running [20, 26]. These motion differences can be noted in the ensemble curves of kinematic data. The mEMG value was lower in the submerged condition than in the non-submerged condition for both movements in the present study, and the iEMG value was higher for the RA muscle and similar for the ES muscle in submerged compared with non-submerged conditions for both movements. This could be due to the longer duration of movement in the submerged condition, as well as additional differences in other muscles. Thus, it was concluded that similar or higher total muscular effort was experienced during submerged chair-based exercise.

Other directions of research are the implementation of pace setting and assessment of the effects of chair surface height and water depth. Previous studies on walking and running classified pace as slow, normal, or fast [13, 21, 27]; however, the present study included exercises that were performed only at a pace that was comfortable for the individual participants. Further investigations involving several pace settings are needed in order to elucidate the detailed features of chair-based/standing exercises in submerged conditions. Different seat heights have been used in previous studies [6– 8, 17, 23, 28]. It can be assumed that water depth will affect muscle activity and movement characteristics, and these should be investigated in future studies. In the present study, no body composition information was collected, which would directly affect the buoyancy level in the submerged condition and could influence EMG and motion kinematics. The results of these future research efforts will contribute to the development of guidelines and protocols for submerged chair-based exercise for rehabilitation of the ability to perform ADLs.

Humans can perform standing and sitting exercises on a chair with safety for acute injuries or failure during exercise in an aquatic environment [29, 30]. Previous studies were conducted on water-based ADL exercise intervention [18, 19]. The results of this study suggest that this form of exercise represents a novel approach to rehabilitate the ability to perform ADLs. One of the most important and distinctive things during exercise, such as ADL movements, in the aquatic environment would be that humans can simulate the movements themselves with a similar joint angular displacement pattern to a real-world situation. This phenomenon has been reported in a walking motion in many previous studies [10, 13]. These exercises can help prevent deterioration of movements in elderly individuals and regain the ability to perform the movements in frail individuals who could not execute the movements by themselves (without any assistance). However, the results of the present study might indicate that the intensity of the exercise was not enough to prevent and regain the movements because basically the muscle activity level in the submerged condition was less than the non-submerged condition. In addition, attention should be paid to the muscle activities of the GAS, BF and RA which were very low through the motion both in the submerged and non-submerged. Future studies will suggest such an effective and detailed intervention program.

In conclusion, the present findings suggest that chair-based exercise with the participant submerged in water reduces muscle activity, especially for sit-to-stand motion which was mostly in according to the previous study, but leads to similar or greater muscular effort for the lower and trunk muscles except for the RF due to the long motion duration in the submerged condition. In addition, the higher muscle activity has been observed especially in BF during stand-to-sit motion in the submerged condition because of buoyancy acted on like water resistance. Moreover, there is flexion of the hip joint and forward inclination of the trunk segment when submerged in water in both standing/sitting motion. Our results present important information which could be beneficial for the exercise and rehabilitation instructors and/or patients with regards to the design and prescription of training regimens.

## References

[1] Burnett DR, Campbell-Kyureghyan NH, Cerrito PB, Quesada PM. Symmetry of ground reaction forces and muscle activity in asymptomatic subjects during walking, sit-to-stand, and stand-to-sit tasks. J Electromyogr Kinesiol. 2011; 21(4): 610–615. 10.1016/j.jelekin.2011.03.006 21493090

[2] Dehail P, Bestaven E, Muller F, Mallet A, Robert B, Bourdel-Marchasson I, et al Kinematic and electromyographic analysis of rising from a chair during a "Sit-to-Walk" task in elderly subjects: role of strength. Clin Biomech. 2007; 22(10): 1096–1103. 10.1016/j.clinbiomech.2007.07.015 17897758

[3] Alexander NB, Schultz AB, Warwick DN. Rising from a chair: effects of age and functional ability on performance biomechanics. J Gerontol. 1991; 46(3): M91–M98. 2030272 10.1093/geronj/46.3.m91

[4] Yamada T, Demura S. Influence of the relative difference in chair seat height according to different lower thigh length on floor reactionforce and lower-limb strength during sit-to-stand movement. J Physiol Anthropol Appl Human Sci. 2004; 23(6): 197–203. 15599063 10.2114/jpa.23.197

[5] Gross MM, Stevenson PJ, Charette SL, Pyka G, Marcus R. Effect of muscle strength and movement speed on the biomechanics of rising from a chair in healthy elderly and young women. Gait Posture. 1998; 8(3): 175–185. 10200407 10.1016/s0966-6362(98)00033-2

[6] Walaszek MC, Ransom AL, Capehart S, Pohl MB, Shapiro R, Bollinger LM. External loading alters trunk kinematics and lower extremity muscle activity in a distribution-specific manner during sitting and rising from a chair. J Electromyogr Kinesiol. 2017; 34: 102–108. 10.1016/j.jelekin.2017.04.005 28460239

[7] Janssen WG, Bussmann JB, Horemans HL, Stam HJ. Analysis and decomposition of accelerometric signals o trunk and thigh obtained during the sit-to-stand movement. Med Biol Eng Comput. 2005; 43(2): 265–272. 15865138 10.1007/BF02345965

[8] Roldán-Jiménez C, Bennett P, Cuesta-Vargas AI. Muscular activity and fatigue in lower-Limb and trunk muscles during different sit-to-stand tests. PLoS One. 2015; 10(10): e0141675 10.1371/journal.pone.0141675 26506612 PMC4624782

[9] Miyoshi T, Shirota T, Yamamoto S, Nakazawa K, Akai M. Functional roles of lower-limb joint moments while walking in water. Clin Biomech. 2005; 20(2): 194–201. 10.1016/j.clinbiomech.2004.10.006 15621325

[10] Miyoshi T, Shirota T, Yamamoto S, Nakazawa K, Akai M. Effect of the walking speed to the lower limb joint angular displacements, joint moments and ground reaction forces during walking in water. Disabil Rehabil. 2004; 26(12): 724–732. 10.1080/09638280410001704313 15204495

[11] Nakazawa K, Yano H, Miyashita M. Ground reaction forces during walking in water In: Miyashita M, Mutoh Y, Richardson AB. editors. Medicine and Science in Aquatic Sports. Med Sport Sci: Basel, Karger; 1994 39: pp. 28–34.

[12] Orselli MI, Duarte M. Joint forces and torques when walking in shallow water. J Biomech. 2011; 44(6): 1170–1175. 10.1016/j.jbiomech.2011.01.017 21334630

[13] Barela AM, Stolf SF, Duarte M. Biomechanical characteristics of adults walking in shallow water and on land. J Electromyogr Kinesiol. 2006; 16(3): 250–256. 10.1016/j.jelekin.2005.06.013 16111894

[14] Kaneda K, Wakabayashi H, Sato D, Uekusa T, Nomura T. Lower extremity muscle activity during deep-water running on self-determined pace. J Electromyogr Kinesiol. 2008; 18(6): 965–972. 10.1016/j.jelekin.2007.04.004 17572106

[15] Fantozzi S, Giovanardi A, Borra D, Gatta G. Gait kinematic analysis in water using wearable inertial magnetic sensors. PLoS One. 2015; 10(9): e0138105 10.1371/journal.pone.0138105 26368131 PMC4569370

[16] Nakazawa K, Yamamoto S, Yano H. Muscle activation patterns during walking in water In: Taguchi K, Igarashi M, Mori S, editors. Vestibular and neural front. Amsterdam: Elsevier Science; 1994 pp. 255–258.

[17] Cuesta-Vargas AI, Cano-Herrera CL, Heywood S. Analysis of the neuromuscular activity during rising from a chair in water and on dry land. J Electromyogr Kinesiol. 2013; 23(6): 1446–1450. 10.1016/j.jelekin.2013.06.001 23834813

[18] Sato D, Kaneda K, Wakabayashi H, Nomura T. The water exercise improves health-related quality of life of frail elderly people at day service facility. Qual Life Res. 2007; 16(10): 1577–1585. 10.1007/s11136-007-9269-2 17952697

[19] Sato D, Kaneda K, Wakabayashi H, Nomura T. Comparison of 2-year effects of once and twice weekly water exercise on activities of daily living ability of community dwelling frail elderly. Arch Gerontol Geriatr. 2009; 49(1): 123–128. 10.1016/j.archger.2008.05.011 18804874

[20] Kaneda K, Sato D, Wakabayashi H, Nomura T. EMG activity of hip and trunk muscles during deep-water running. J Electromyogr Kinesiol. 2009; 19(6): 1064–1070. 10.1016/j.jelekin.2008.11.001 19097917

[21] Kaneda K, Ohgi Y, McKean M, Burkett B. Measuring and classifying land-based and water-based daily living activities using inertial sensors. Proceedings. 2018; 2 10.3390/proceedings2060298

[22] Perotto A, Morrison D, Delagi E, Iazzetti J. Anatomical guide for the electromyographer: The limbs and trunk. 3rd ed Springfield: Thomas; 1994.

[23] Pai YC, Rogers MW. Speed variation and resultant joint torques during sit-to-stand. Arch Phys Med Rehabil. 1991; 72(11): 881–885. 1929805 10.1016/0003-9993(91)90004-3

[24] Millington PJ, Myklebust BM, Shambes GM. Biomechanical analysis of the sit-to-stand motion in elderly persons. Arch Phys Med Rehabil. 1992; 73(7): 609–617. 1622314

[25] Clément G, Gurfinkel VS, Lestienne F, Lipshits MI, Popov KE. Adaptation of postural control to weightlessness. Exp Brain Res. 1984; 57(1): 61–72. 10.1007/bf00231132 6519230

[26] Moening D, Scheidt A, Shepardson L, Davis GJ. Biomechanical comparison of water running and treadmill running. Isokinet Exerc Sci. 1993; 3(4): 207–215.

[27] Killgore GL. Deep-water running: a practical review of the literature with an emphasis on biomechanics. Phys Sportsmed. 2012; 40(1): 116–126. 10.3810/psm.2012.02.1958 22508258

[28] Goulart FR, Valls-Solé J. Patterned electromyographic activity in the sit-to-stand movement. Clin Neurophysiol. 1999; 110(9): 1634–1640. 10479031 10.1016/s1388-2457(99)00109-1

[29] Devereux K, Robertson D, Briffa NK. Effects of a water-based program women 65 years and over: A randomized controlled trial. Aust J Physiother. 2005; 51(2): 102–108. 15924512 10.1016/s0004-9514(05)70038-6

[30] Forwood MR, Larsen JA. Exercise recommendations for osteoporosis. A position statement of the Australian and New Zealand Bone and Mineral Society. Aust Fam Physician. 2000; 29(8): 761–764. 10958022

